# PPARα Genetic Deletion Reveals Global Transcriptional Changes in the Brain and Exacerbates Cerebral Infarction in a Mouse Model of Stroke

**DOI:** 10.3390/ijms26094082

**Published:** 2025-04-25

**Authors:** Milton H. Hamblin, Austin C. Boese, Rabi Murad, Jean-Pyo Lee

**Affiliations:** 1 Division of Biomedical Sciences, School of Medicine, University of California Riverside, Riverside, CA 92521, USA; 2 Health Sciences Center, Tulane University, New Orleans, LA 70112, USA; 3 School of Medicine, Emory University, Atlanta, GA 30322, USA; austin.c.boese@emory.edu; 4 Bioinformatics, Sanford Burnham Prebys Medical Discovery Institute, La Jolla, CA 92037, USA; rmurad@sbpdiscovery.org; 5 Department of Physiology, Tulane University School of Medicine, New Orleans, LA 70112, USA

**Keywords:** ischemic stroke, peroxisome proliferator-activated receptor alpha, RNA sequencing

## Abstract

Ischemic stroke is a leading cause of death and disability worldwide. Currently, there is an unmet clinical need for pharmacological treatments that can improve ischemic stroke outcomes. In this study, we investigated the role of brain peroxisome proliferator-activated receptor alpha (PPARα) in ischemic stroke pathophysiology. We used a well-established model of cerebral ischemia in PPARα transgenic mice and conducted the RNA sequencing (RNA-seq) of mouse stroke brains harvested 48 h post-middle cerebral artery occlusion (MCAO). PPARα knockout (KO) increased brain infarct size following stroke, indicating a protective role of PPARα in brain ischemia. Our RNA-seq analysis showed that PPARα KO altered the expression of genes in mouse brains with known roles in ischemic stroke pathophysiology. We also identified many other differentially expressed genes (DEGs) upon the loss of PPARα that correlated with increased infarct size in our stroke model. Gene set enrichment analysis (GSEA) and Gene Ontology (GO) analysis revealed the upregulation of gene signatures for the positive regulation of leukocyte proliferation, apoptotic processes, acute-phase response, and cellular component disassembly in mouse stroke brains with PPARα KO. In addition, pathway analysis of our RNA-seq data revealed that TNFα signaling, IL6/STAT3 signaling, and epithelial–mesenchymal transition (EMT) gene signatures were increased in PPARα KO stroke brains. Our study highlights PPARα as an attractive drug target for ischemic stroke due to its transcriptional regulation of inflammation-, apoptosis-, and EMT-related genes in brain tissue following ischemia.

## 1. Introduction

Ischemic stroke is characterized by the sudden obstruction of blood flow to the brain and remains a leading cause of mortality and long-term disability worldwide. In 2021, stroke accounted for approximately 1 of every 21 deaths in the United States [1]. To date, the only FDA-approved acute treatments for ischemic stroke are thrombolysis with tissue plasminogen activator (tPA) and mechanical thrombectomy. The therapeutic window for these interventions is narrow, and tPA is known to increase the risk of hemorrhagic transformation in stroke patients if administered too late. Thus, many ischemic stroke patients experience irreversible brain damage and long-term disability. There is an unmet clinical need for new pharmacological therapies that can reduce infarct size, limit neurological deficits, and promote recovery in stroke patients. The pathophysiology of ischemic stroke involves cellular energy failure, neural excitotoxicity, oxidative stress, inflammation, and ultimately cell death [2,3,4,5]. Thus, druggable targets that can modulate several of these pathways are attractive candidates for stroke therapy.

Peroxisome proliferator-activated receptor alpha (PPARα) is a member of the nuclear hormone receptor superfamily and plays a central role in lipid metabolism, energy homeostasis, and inflammation [6]. When bound to its ligands, PPARα traffics to the nucleus and functions as a transcription factor to regulate the expression of genes involved in fatty acid oxidation and inflammation [7]. Previous research on PPARα characterized its physiological role in the vasculature, where it regulates lipid metabolism, inflammation, and endothelial function [8,9]. In fact, PPARα agonists such as fenofibrate, gemfibrozil, and bezafibrate are used in the clinic to manage cardiovascular disease by dampening inflammation, reducing triglyceride levels, and increasing beneficial HDL cholesterol [10].

Accumulating evidence suggests that PPARα also plays a neuroprotective role in the brain. Several studies report that pretreatment with PPARα agonists like fenofibrate increases the activity of antioxidant enzymes, decreases oxidative stress, and improves outcomes in rodent models of ischemic stroke [11,12,13]. Some preclinical studies suggest that treatment with PPARα agonists benefits stroke outcome by eliciting anti-inflammatory effects in the CNS and the periphery [11,12,14,15,16], while other studies report that PPARα activation reduces endothelial dysfunction and blood–brain barrier (BBB) breakdown after stroke [17,18]. Altogether, PPARα activation may protect against ischemic stroke through the modulation of several pathophysiological pathways such as oxidative stress, inflammation, and blood vessel dysfunction. However, the detailed transcriptional targets that PPARα regulates in the brain vasculature during ischemia remain largely unknown. In this study, we modeled ischemic stroke in wild-type (WT) and PPARα genetic knockout (KO) mice and utilized the RNA-sequencing of ipsilesional hemisphere brain tissue harvested 48 hours (h) post-stroke to characterize genes and transcriptional pathways associated with PPARα. Our study is the first to use global CNS transcriptome profiling to investigate the role of PPARα in the subacute phase of ischemic stroke.

## 2. Results

### 2.1. PPARα KO Increases Infarct Volume in Mouse Stroke Brains

**Figure 1:**
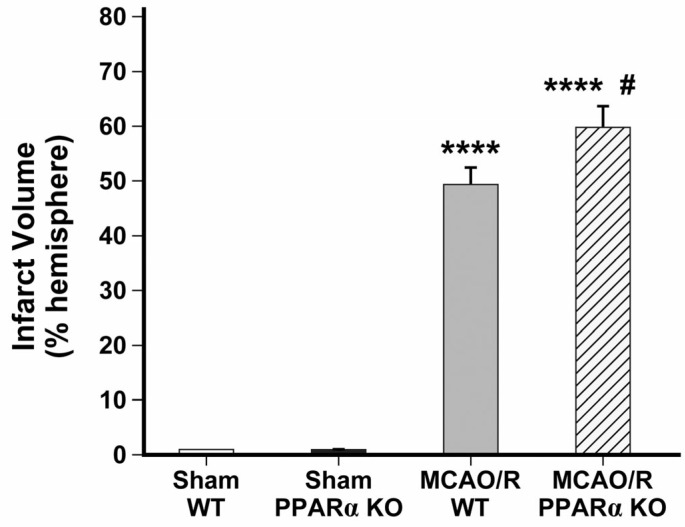
PPARα KO increases stroke infarct volume. Infarct volume calculated by TTC staining of male mouse brains harvested 48 h post-MCAO (PPARα KO vs. WT). **** *p* < 0.0001 vs. sham; # *p* < 0.05 vs. MCAO/R WT. (*n* = 10, sham WT; *n* = 9, sham PPARα KO; *n* = 11, MCAO/R WT; *n* = 9, MCAO/R PPARα KO). Data are presented as mean ± SEM. MCAO/R, middle cerebral artery occlusion with reperfusion.

**Figure A1:**
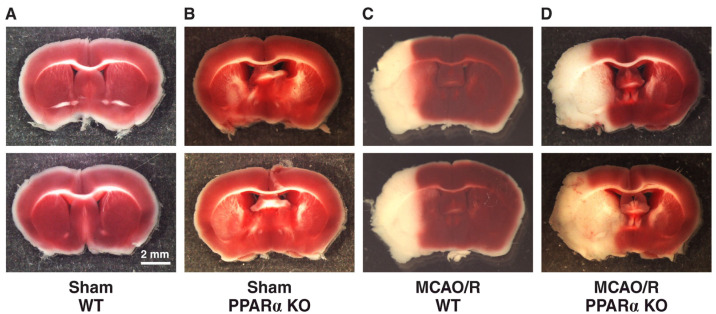
PPARα knockout increases infarct volume after stroke. TTC-stained mouse brain sections 48 h post-stroke show infarcted tissue (white) and viable tissue (red). Ischemic damage is shown in both the caudoputamen and cortex. Panels show representative coronal sections: (**A**) Sham WT, (**B**) Sham PPARα KO, (**C**) MCAO/R WT, and (**D**) MCAO/R PPARα KO. Each panel shows sections from two different brains. MCAO/R, middle cerebral artery occlusion with reperfusion. Scale bar = 2 mm.

Treatment with PPARα agonists has beneficial effects on the vasculature, but PPARα’s role in the context of ischemic stroke is not well understood. We assessed the effects of PPARα genetic deletion on infarct volume in mouse stroke brains. We used the TTC staining of brain sections to quantify the ischemic lesion volume (percentage of the brain hemisphere) 48 h after middle cerebral artery occlusion followed by reperfusion (MCAO/R). Compared with brains from MCAO/R WT mice, brains from MCAO/R PPARα KO mice had significantly larger infarct sizes, indicating greater stroke severity (Figure A1). The mean infarct volume of the ipsilesional hemisphere in MCAO/R WT stroke brains was 49.4 ± 2.89% (**** *p* < 0.0001 vs. sham). In MCAO/R PPARα KO brains, the mean infarct volume was 59.74 ± 3.57% (**** *p* < 0.0001 vs. sham), which was larger than in the MCAO/R WT group (# *p* < 0.05 vs. MCAO/R WT) (Figure 1). These results suggest that the loss of PPARα worsens ischemic brain injury, indicating a protective role of PPARα in brain ischemia.

### 2.2. Genetic Deletion of PPARα Elicits Global Transcriptional Changes in Mouse Brains After Stroke

**Figure 2:**
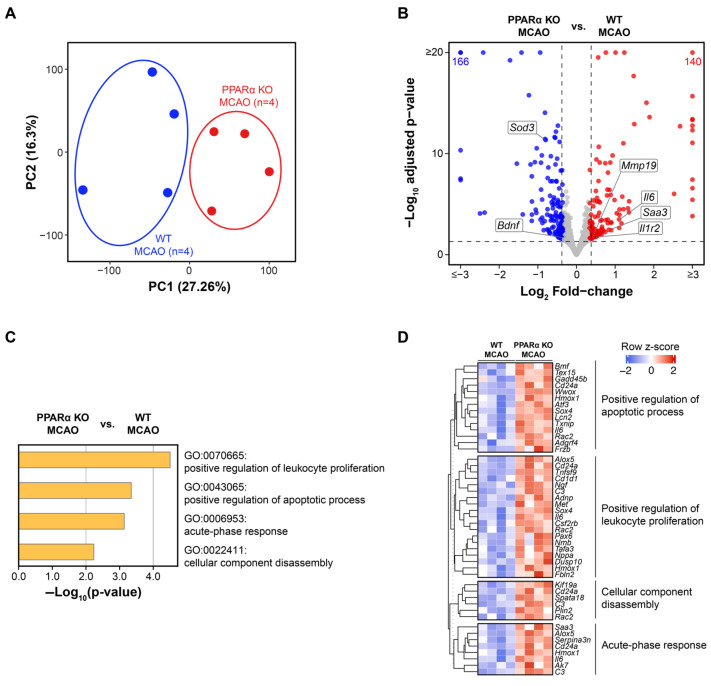
PPARα KO elicits global transcriptional changes in the brains of male mice by 48 h post-stroke. (**A**) Principal component analysis (PCA) of transcriptomes from PPARα KO MCAO (*n* = 4, red) and WT MCAO (*n* = 4, blue) mouse brains harvested at 48 h post-stroke. Each dot represents a biological replicate of an RNA-seq sample, and principal component 1 (PC1; 27.26% variance) splits the samples according to PPARα genetic status. (**B**) Volcano plot of differentially expressed genes (DEGs) between PPARα KO MCAO and WT MCAO samples. Genes downregulated by PPARα KO in stroke brains are colored blue. Genes upregulated by PPARα deletion in stroke brains are colored red. Representative DEGs are labeled in black. (**C**) Gene Ontology (GO) analysis results of DEGs between PPARα KO MCAO and WT MCAO brains. (**D**) Heatmaps of gene sets corresponding to positive regulation of apoptotic process, positive regulation of leukocyte proliferation, cellular component disassembly, and acute-phase response. Row-wise z-scores were computed using transcripts per million (TPM). Core enriched genes of interest in each gene set are labeled in black. Genes are clustered based on their expression patterns, with red indicating higher expression and blue indicating lower expression relative to the mean. MCAO, middle cerebral artery occlusion with reperfusion.

We performed RNA-seq on ipsilesional hemisphere brain tissue harvested from mice at 48 h post-MCAO to investigate the effects of PPARα KO on gene expression during the subacute stroke phase. We further analyzed differentially expressed genes (DEGs) between PPARα KO brains and WT brains to better characterize molecular pathways through which the loss of PPARα exacerbated ischemic stroke. We first compared the overall transcriptomes of PPARα KO mouse stroke brains with those of WT stroke brains (PPARα KO MCAO vs. WT MCAO). Principal component analysis (PCA) showed the clear separation of PPARα KO MCAO and WT MCAO brain transcriptomes along PC1, which accounted for the greatest variability (27.26%) in the data (Figure 2A). These PCA results suggested widespread transcriptomic differences in brain tissue due to PPARα deletion. Overall, we found 166 downregulated and 140 upregulated genes in PPARα KO stroke brains with a fold change >1.5 in either direction (FDR < 0.05) (Figure 2B). PPARα KO brains had upregulated expression of several genes already implicated in neural injury following stroke such as *Mmp19*, *Il6*, *Saa3*, and *Il1r2* (Figure 2B). We also observed the downregulation of genes well known to be neuroprotective following stroke such as *Sod3* and *Bdnf* (Figure 2B). To further characterize cellular processes affected in stroke brains by the genetic deletion of PPARα, we conducted gene set enrichment analysis (GSEA) on DEGs between PPARα KO MCAO and WT MCAO brain transcriptomes. In PPARα KO stroke brains, we observed the enrichment of Gene Ontology (GO) Biological Processes and Molecular Functions for the following gene sets: the positive regulation of leukocyte proliferation (−log_10_ (*p*-value) = 4.501, Enrichment = 7.871), positive regulation of apoptotic process (−log_10_ (*p*-value) = 3.345, Enrichment = 3.349), acute-phase response (−log_10_ (*p*-value) = 3.137, Enrichment = 16.962), and cellular component disassembly (−log_10_ (*p*-value) = 2.235, Enrichment = 3.699) (Figure 2C). Heatmaps comparing the expression of key genes for each respective GO pathway between PPARα KO MCAO brains and WT MCAO brains are presented in Figure 2D.

### 2.3. PPARα Deletion Alters Gene Expression Signatures for TNFα Signaling, IL6 Signaling, and Epithelial–Mesenchymal Transition (EMT) in Stroke Brains

**Figure 3:**
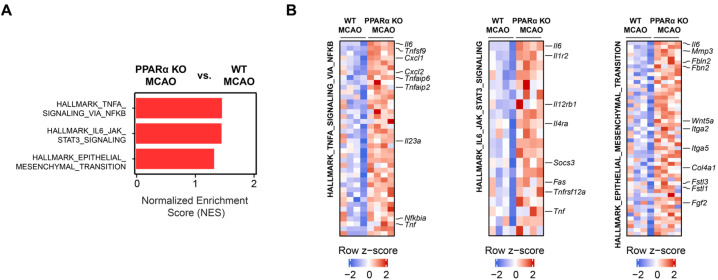
PPARα KO transcriptionally upregulates EMT, TNFα signaling, and IL6/JAK/STAT3 signaling in the subacute stroke phase. (**A**) Normalized enrichment scores (NESs) comparing PPARα KO MCAO (*n* = 4) vs. WT MCAO (*n* = 4) brain transcriptomes for HALLMARK_TNFA_VIA_NFKB, HALLMARK_IL6_JAK_STAT3_SIGNALING, and HALLMARK_EPITHELIAL_MESENCHYMAL_TRANSITION gene set signatures. *p* < 0.05. (**B**) Expression heat maps of core enriched genes from panel A hallmark gene sets. Row-wise z-scores were computed using normalized transcripts per million (TPM) expression values. Core enriched genes of interest in each gene set are labeled in black. MCAO, middle cerebral artery occlusion with reperfusion.

**Figure A2:**
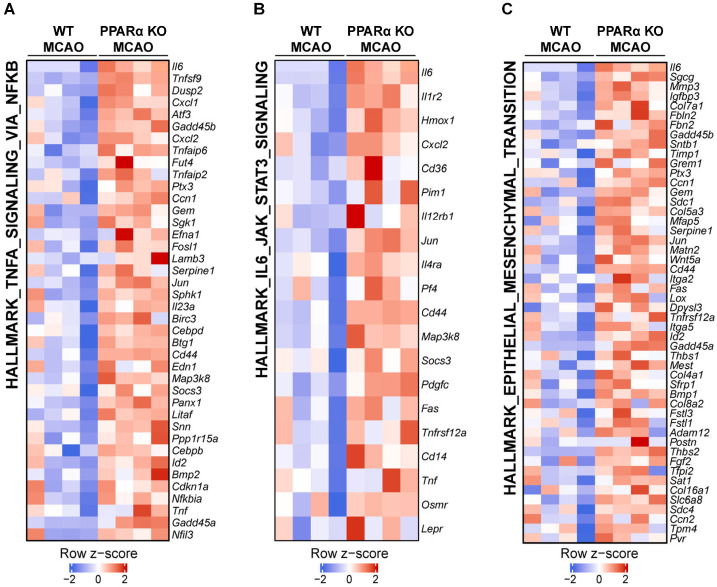
Clustering analysis of mouse Hallmark gene sets related to (**A**) TNFα signaling via NFKB, (**B**) IL6/JAK/STAT3 signaling, and (**C**) Epithelial–Mesenchymal Transition in PPARα KO MCAO (*n* = 4) compared with WT MCAO (*n* = 4) mouse brains harvested 48 h post-stroke. Row-wise z-scores were computed using transcripts per million (TPM). MCAO, middle cerebral artery occlusion with reperfusion.

We further analyzed our RNA-seq data to identify enriched or depleted cellular pathways resulting from PPARα KO in mouse stroke brains. GSEA revealed positive normalized enrichment scores (NES), indicating the upregulation of Hallmark gene sets for TNFα signaling via NFKB (NES = 1.442), IL6/JAK/STAT3 signaling (NES = 1.435), and epithelial–mesenchymal transition (EMT) (NES = 1.308) in the brains of PPARα KO MCAO mice (Figure 3A). The positive enrichment scores across these pathways indicate their upregulation in ischemic brain tissue from the loss of PPARα and point to a potential protective or modulatory effect of PPARα against ischemia-induced pathological changes in gene expression. Key TNFα signaling-related genes that were upregulated in PPARα KO MCAO brains included *Il6*, *Tnfsf9*, *Cxcl1*, *Cxcl2*, *Tnfaip6*, *Tnfaip2*, *Il23a*, *Nfkbia*, and *Tnf*. Key genes related to IL6/JAK/STAT3 signaling that were upregulated in PPARα KO MCAO brains included *Il6*, *Il1r2*, *Il12rb1*, *Il4ra*, *Socs3*, *Fas*, *Tnfrsf12a*, and *Tnf*. Key EMT-related genes upregulated in PPARα KO MCAO brains included *Il6*, *Mmp3*, *Fbln2*, *Fbn2*, *Wnt5a*, *Itga2*, *Itga5*, *Col4a1*, *Fstl3*, *Fstl1*, and *Fgf2* (Figure 3B and Figure A2). Clinical evidence shows that blood and cerebrospinal levels of IL6 are significantly elevated in patients following stroke [19,20,21]; the JAK/STAT3 pathway is highly implicated in post-stroke inflammation by inducing the transcription of inflammatory mediators like cytokines and chemokines [22]. Similarly, TNFα levels increase in the brain and blood following stroke and increase neuroinflammation [23,24]. Previous research links TNFα levels to worse stroke patient outcomes, and preclinical studies demonstrate that TNFα signaling recruits immune cells to the CNS, disrupts BBB function, and promotes brain cell apoptosis during ischemic stroke [21,25]. In addition to inflammation, epithelial-to-mesenchymal transition (EMT) and endothelial-to-mesenchymal transition (EndMT) are processes whereby epithelial or endothelial cells lose their typical morphology and function and gain mesenchymal properties. Following the acute phase of stroke, the inflammatory and hypoxic environment in the brain can trigger EMT/EndMT in cells that form the BBB, which promotes its breakdown, thereby exacerbating edema and allowing the infiltration of immune cells into the brain [26]. The resulting neuroinflammation can worsen tissue damage and impede stroke recovery. Additionally, EndMT may promote fibrosis in the brain vasculature, further impairing blood flow and contributing to long-term injury following stroke [27]. Overall, our results suggest that PPARα signaling may blunt signaling pathways that promote inflammation and BBB breakdown in the subacute stroke phase to reduce the infarct volume.

### 2.4. Ingenuity Pathway Analysis Reveals TNFα as an Upstream Regulator Affected by PPARα KO in Mouse Stroke Brains

**Figure 4:**
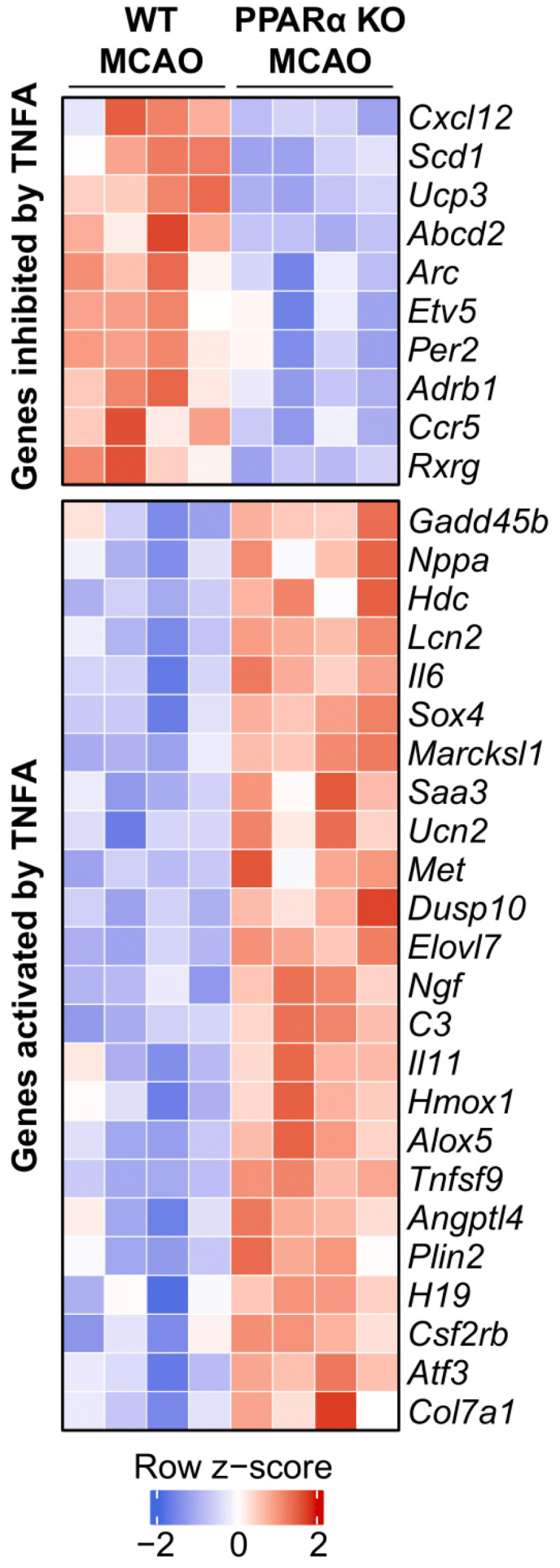
PPARα knockout alters TNFα-mediated gene expression in mouse stroke brains. Heatmap of genes downstream of TNFα signaling differentially expressed in PPARα KO MCAO (*n* = 4) vs. WT MCAO (*n* = 4) brains. Genes in blue are downregulated and genes in red are upregulated in PPARα KO stroke brains compared with WT stroke brains. TNFα signaling activity is inferred to be increased based on the expression patterns of its downstream targets. Row-wise z-scores were computed using normalized transcripts per million (TPM) expression values. MCAO, middle cerebral artery occlusion with reperfusion.

**Figure A3:**
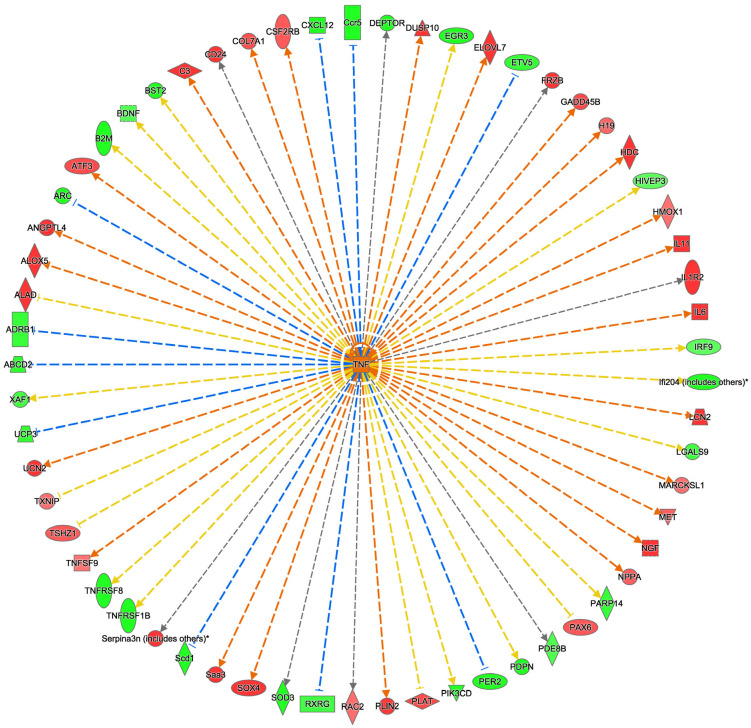
Predicted activation of TNFα as an upstream regulator in PPARα KO stroke brains. Upstream Regulator Analysis in Ingenuity Pathway Analysis (IPA) predicts TNFα activation based on differentially expressed target genes in PPARα KO MCAO (*n* = 4) vs. WT MCAO (*n* = 4) mouse brains 48 h post-stroke. Upregulated targets (red) and downregulated targets (green) indicate TNFα-driven transcriptional changes in PPARα KO stroke vs. WT stroke. TNFα is predicted to be activated (center, orange) based on the collective expression patterns of its downstream targets. (includes others)* indicates that the regulator is part of a broader group or network. MCAO, middle cerebral artery occlusion with reperfusion.

We conducted Ingenuity Pathway Analysis (IPA) on our RNA-seq data to further interrogate the cellular pathways affected by PPARα KO in mouse stroke brains. Similar to GSEA, IPA revealed significant transcriptional reprogramming of TNFα signaling gene signatures in PPARα KO stroke brains 48 h post-MCAO. We observed decreased expression of several genes known to be suppressed by TNFα in PPARα KO brains such as *Cxcl12*, *Scd1*, *Ucp3*, *Abcd2*, *Arc*, *Etv5*, *Per2*, *Adrb1*, *Ccr5*, and *Rxrg* (Figure 4). In agreement, we also observed an increased expression profile for many genes known to be upregulated by TNFα in stroke brains from PPARα KO mice. Specifically, PPARα KO led to increased expression levels of *Gadd45b*, *Nppa*, *Hdc*, *Lcn2*, *Il6*, *Sox4*, *Marcksl1*, *Saa3*, *Ucn2*, *Met*, *Dusp10*, *Elovl7*, *Ngf*, *C3*, *Il11*, *Hmox1*, *Alox5*, *Tnfsf9*, *Angptl4*, *Plin2*, *H19*, *Csf2rb*, *Atf3*, and *Col7a1* following stroke (Figure 4). The overall expression pattern of the downstream targets indicates that TNFα is an activated upstream regulator in PPARα KO stroke brains compared with WT stroke brains (Figure A3). The predominance of upregulated downstream targets (red nodes) suggests that TNFα drives a pro-inflammatory transcriptional program following brain ischemia and that PPARα normally suppresses this response.

## 3. Discussion

Ischemic stroke pathophysiology is complex and involves blood–brain barrier (BBB) breakdown, oxidative stress, neuroinflammation, and the ultimate death of CNS resident cells [28]. Thus, future pharmacological therapies for stroke should prioritize druggable targets that can limit brain tissue damage through multiple mechanisms. PPARα is well known for its role in vascular function, and PPARα agonists such as clofibrate, fenofibrate, and gemfibrozil are used in the clinic as antilipemic agents to manage cardiovascular disease. PPARα is expressed in endothelial cells (ECs) [29], vascular smooth muscle cells (VSMCs) [30], and immune cells such as monocytes and macrophages [31], where it functions as a ligand-activated transcription factor to regulate gene expression. Ligand-activated PPARα dimerizes with the retinoid X receptor (RXR) [7] and associates with PPAR response elements (PPREs) in the promoter regions of genes to regulate their transcription. PPARα can also repress gene expression by binding and inhibiting other transcription factors like nuclear factor kappa-light-chain-enhancer of activated B cells (NFκB) and activator protein-1 (AP1) [32,33]. The anti-inflammatory effects of fibrates are mostly attributed to the PPARα-mediated suppression of inflammatory gene expression in vascular and immune cells.

The activation of PPARα improves outcomes in rodent models of ischemic stroke. In Apolipoprotein E (ApoE)-deficient mice, pretreatment with fenofibrate for 14 days (d) prior to MCAO resulted in reduced infarct size and increased antioxidant activity from superoxide dismutase, glutathione reductase, glutathione peroxidase, and glutathione S-transferase [11]. Another study using male Wistar rats reported that 14 d pretreatment with fenofibrate reduced the infarct volume and markers of both neuroinflammation and oxidative stress following ischemic stroke [12]. Other researchers reported that daily treatment with fenofibrate for 7 d prior to 1 h MCAO resulted in upregulated gene expression of all three superoxide dismutase (SOD) isoforms in the brain microvasculature and reduced levels of reactive oxygen species (ROS) and oxidative damage in adult male mice [13]. Together, these studies highlight PPARα agonists as potential pharmacological candidates for stroke therapy. However, the transcriptional pathways that PPARα regulates to protect the brain from ischemia are not yet fully understood.

To address these gaps in our knowledge, we performed the RNA-sequencing of ipsilesional hemisphere brain tissue harvested from WT and PPARα KO adult male mice at 48 h post-MCAO. Through bioinformatic analysis on our RNA-sequencing data, we were able to identify several PPARα KO-regulated transcriptional pathways associated with increased stroke infarct sizes. Our results agree with previous studies that implicate PPARα in regulating superoxide dismutase enzyme expression. In our study, PPARα KO significantly downregulated the brain expression of superoxide dismutase 3 (*Sod3*) by 48 h post-MCAO. SOD3 is an extracellular antioxidant enzyme that protects against ischemic stroke by scavenging ROS produced during ischemia–reperfusion injury [34]. Increased SOD3 is also associated with reduced BBB damage, smaller infarct volumes, and improved neurological outcomes following ischemic stroke [35]. Thus, our data also suggest a link between PPARα and the expression of antioxidant genes post-stroke. We observed a decreased expression of other well-known neuroprotective genes like brain-derived neurotrophic factor (*Bdnf*) 48 h post-MCAO in PPARα KO mouse brains. BDNF promotes the survival and growth of neurons, enhances synaptic plasticity, and promotes the repair and regeneration of brain tissue following ischemic injury [36]. Overall, our RNA-seq analysis identified several well-established neuroprotective genes regulated by PPARα that may explain the increased infarct size observed in our PPARα KO mice.

Interestingly, we also observed that the genetic deletion of PPARα increased the expression of several genes that are strongly implicated in ischemic stroke pathophysiology. This highlights a potential neuroprotective role of PPARα-mediated gene repression in the brain following stroke. Specifically, we observed the upregulation of several inflammatory genes such as *C3*, *Il6*, and *Il1r2* 48 h post-MCAO in PPARα KO mouse brains, which correlated with increased infarct size. Gene Ontology (GO) analysis also identified the enrichment of gene signatures for the positive regulation of leukocyte proliferation and the acute-phase response in the brains of PPARα KO mice. Thus, our data agree with previous studies in which fenofibrate treatment reduced markers of neuroinflammation in rodent models of stroke. Of note, IL1 signaling and C3 protein levels are known to be increased following stroke and correlate with worse clinical outcomes in ischemic stroke patients [37,38,39]. Similarly, blood and cerebrospinal fluid levels of IL6 increase following stroke and correlate with stroke severity and worse stroke outcomes [19,20,21]. Specifically, IL6 signaling through the Janus kinase (JAK) and signal transducer and activator of transcription 3 (STAT3) pathway plays a major role in post-stroke inflammatory responses through the transcription of various inflammatory genes [22]. In the acute phase of stroke, high levels of IL6 can contribute to an inflammatory response by activating immune cells and promoting the release of other pro-inflammatory cytokines. Further bioinformatic analysis of our RNA-seq data revealed the upregulation of other genes in the IL6/JAK/STAT3 expression signatures in mouse stroke brains upon the loss of PPARα, as well. For instance, we found that PPARα KO increased the expression of *Il12rb1* (Interleukin-12 receptor beta 1) by 48 h post-stroke; signaling through IL12Rβ1 promotes interferon-gamma (IFNγ) production [40], which worsens neuroinflammation post-stroke by activating immune cells to produce more inflammatory cytokines and chemokines. We also noted a higher expression of *Socs3* (suppressor of cytokine signaling 3) in PPARα KO brains after stroke. Reducing SOCS3 levels in the brain is reported to polarize macrophages towards the M2 phenotype and promote the resolution of post-stroke inflammation [41]. Therefore, PPARα likely restrains the expression of genes linked to IL6/JAK/STAT3 signaling to prevent rampant neuroinflammation in the subacute stroke phase.

We also observed the upregulation of a pro-inflammatory tumor necrosis factor alpha (TNFα) gene signature in the brains of PPARα KO mice following stroke. Key genes in this signature included *Tnfsf9*, *Cxcl1*, *Cxcl2*, *Il23a*, *Nfkbia*, and *Tnf*. TNFα is a pro-inflammatory cytokine that is rapidly upregulated in the brain following ischemic stroke and promotes the recruitment of immune cells to the site of injury [24]. In addition, TNFα can induce apoptosis in neurons and other brain cells and contribute to BBB dysfunction, thus further increasing tissue damage after stroke [23,24]. CXCL1/CXCL2 are chemokines that promote the migration of neutrophils and other immune cells to the infarcted area, which causes further tissue loss [25,42]. Additionally, TNFSF9 (tumor necrosis factor superfamily member 9) is upregulated in response to cerebral ischemia–reperfusion injury and can exacerbate inflammation and cell death by promoting ferroptosis and apoptosis in brain microvascular endothelial cells [43]. Of note, elevated TNFα levels are linked to worse outcomes in stroke patients, and overwhelming preclinical evidence shows that TNFα signaling increases brain damage following stroke through sustained neuroinflammation, the disruption of BBB function, and the promotion of neural apoptosis [21,25]. Thus, PPARα likely curtails neuroinflammation following stroke through the regulation of TNFα signaling-related genes.

As mentioned previously, BBB disruption and brain cell death coincide with neuroinflammation and are key hallmarks of stroke that lead to long-term neurological disability. Reperfusion injury following stroke damages the BBB and ultimately results in the loss of tight junctions between endothelial cells that form this crucial neurovascular structure. EMT/EndMT (epithelial–mesenchymal transition/endothelial–mesenchymal transition) is a process whereby endothelial cells lose their characteristics and gain mesenchymal properties. In the context of stroke, EMT/EndMT contributes to BBB breakdown by promoting endothelial cell dysfunction and increasing vascular permeability [27]. Other processes such as apoptosis, necroptosis, and the destruction of cellular components that form tight junctions can cause BBB breakdown and worsen stroke outcomes [44,45]. The apoptosis of neurons, glial cells, and brain microvascular cells post-stroke can occur intrinsically due to mitochondrial energy failure, oxidative stress, and calcium imbalance [46]. Immediately following stroke, the acute-phase response is activated and initiates an innate immune response to the ischemic lesion to clear dead and damaged cells. Thus, brain cell loss also occurs through the extrinsic apoptotic pathway mediated by death receptor signaling from immune cells that infiltrate the brain following stroke [46]. While this plays a role in wound healing and repair, prolonged activation and neuroinflammation contribute to extensive brain cell loss and worsen stroke outcomes. Interestingly, we observed the transcriptional enrichment of several of these pathways in the brains of our PPARα KO mice during the subacute stroke phase. Specifically, the GO analysis of our RNA-seq data revealed that the loss of PPARα in the brain led to the enrichment of gene sets related to the acute-phase response, apoptosis, and cellular component disassembly 48 h post-MCAO. Thus, in addition to limiting neuroinflammation, PPARα activation likely protects the brain following stroke by also modulating genes involved in apoptosis, EMT, and the disassembly of cellular components.

PPARα functions as a ligand-activated transcription factor, and we identified hundreds of genes that were differentially regulated in the post-stroke brain upon its genetic deletion. Although we delineated several transcriptional pathways modulated by PPARα, it remains unknown which of these genes are directly regulated by PPARα’s transcription factor activity at regulatory elements upstream of transcription start sites or whether PPARα modulates the expression of some of our identified genes in an indirect manner by modulating the expression of other upstream regulators and transcription factors. Although previous studies in animal models of stroke have reported neuroprotective effects from treatment with PPARα agonists like fenofibrate, further genetic approaches are needed to corroborate these findings. A complementary positive approach using gain-of-function studies with constitutive PPARα signaling in different types of brain cells will help to validate our findings and identify additional PPARα-regulated pathways that may benefit stroke outcomes. Future studies on the precise functions of PPARα in the brain during ischemic stroke will benefit from the use of epigenetic profiling in parallel with RNA-seq or other gene expression assays, to determine the genes that are directly regulated by PPARα’s transcription factor binding activity.

Previous studies using transgenic PPARα KO mice confirmed that PPARα regulates lipid metabolism and cellular energy balance [47,48]. These disruptions may lead to conditions like insulin resistance, obesity, or fatty liver disease, all of which can negatively impact the body’s response to injury and ability to recover. The pathophysiology of ischemic stroke involves energy failure resulting from oxygen and glucose deprivation. Thus, any alterations to cellular metabolism in the CNS are likely to impact stroke outcomes. Future studies using PPARα transgenic mice to model ischemic stroke may consider monitoring physiological and biochemical variables, such as lipid profiles, glucose levels, and liver function, to better assess how the metabolic consequences of PPARα modulation may affect stroke pathophysiology. In addition, we used male transgenic mice for our rodent model of stroke. Although we observed PPARα KO-associated modulation of several pathways linked to increased infarct volume in male stroke brains, it is possible that PPARα modulates transcriptional pathways in the CNS differently between males and females. Future studies would benefit from a comparison of the effect of PPARα KO on stroke outcomes in both male and female transgenic mice and the use of ChIP-seq and proteomics to interrogate whether PPARα cooperates with androgen and estrogen receptors in the nucleus of brain cells to influence gene expression differentially according to biological sex. Nevertheless, our study highlights PPARα as an attractive candidate for pharmacological stroke therapy that warrants further research.

## 4. Materials and Methods

### 4.1. Animals

Transgenic PPARα KO mice and littermate controls (8–12 weeks) were obtained from Jackson Laboratories (B6; 129S4-*Ppara ^tm1Gonz^*/J, Bar Harbor, ME, USA). Mice were kept at 18–22 °C on a 12 h light–dark cycle. Mice were given food and water ad libitum.

### 4.2. Mouse Model of Stroke

Tulane University (New Orleans, LA, USA) and the University of California, Riverside (Riverside, CA, USA) Institutional Animal Care and Use Committees reviewed and approved animal use for this study. Animals were cared for and treated according to the guidelines of Tulane University and UCR animal protocols, the National Institutes of Health Guide for the Care and Use of Laboratory Animals, and the American Veterinary Medical Association.

We used middle cerebral artery occlusion followed by reperfusion to model transient focal cerebral ischemia in mice [49]. Stroke surgery was performed as previously published [50,51,52,53]. MCAO was performed for 1 h using a 6-0 nylon monofilament (Doccol Corporation, Sharon, MA, USA), and then the filament was removed to allow for reperfusion. For sham surgical controls, mouse cerebral arteries were inserted with the filament followed by immediate removal. Successful MCAO was defined as a reduction in regional cerebral blood flow (rCBF) by >80% and was assessed with a transcranial laser Doppler (Perimed Inc., Las Vegas, NV, USA). Blood reperfusion to >90% of baseline rCBF was the criterion used to determine successful post-MCAO/R recovery.

### 4.3. Infarct Volume Quantification

Brain ischemic lesions were visualized by sectioning mouse brains and staining with 2,3,5-triphenyl tetrazolium chloride (TTC, Sigma-Aldrich, St. Louis, MO, USA). Mouse brains were harvested 48 h after MCAO and sliced into 1 mm coronal sections. Brain sections were incubated in 2% TTC solution as previously described [50,51,52]. ImageJ v4 software (National Institutes of Health, Bethesda, MD, USA) was used to calculate the infarct area of stained brain sections. The cerebral infarct volume was calculated as a percentage of the contralateral hemisphere using the following formula: [volume of contralateral hemisphere − (volume of total ipsilesional hemisphere − volume of infarct area)]/volume of contralateral hemisphere × 100. This calculation method was able to compensate for post-stroke edema [54].

### 4.4. RNA Sequencing (RNA-Seq)

Cellular mRNA was isolated using the NEBNext^®^ Poly(A) mRNA Magnetic Isolation Kit. The NEBNext^®^ Ultra™ Directional RNA Library Prep Kit for Illumina^®^ (NEB, Ipswich, MA, USA) was used to construct libraries for RNA sequencing. Libraries were pooled and sequenced as single-end 75 bp on an Illumina NextSeq 500 sequencer with a sequencing depth of 18–30 million reads.

#### 4.4.1. RNA-Seq Data Processing

RNA-seq read trimming, alignment, gene quantification, normalization, and QC were performed using the nf-core rnaseq pipeline v3.14.0 [55] with the --aligner star_rsem parameter. The Mus musculus (house mouse) genome assembly GRCm38 (mm10) and Gencode vM25 gene annotations [56] were used for alignment.

#### 4.4.2. Quality Control and Assurance (QC/QA) of RNA-Seq Data

RNA sequencing alignment and quantification quality was assessed by FastQC and MultiQC using the nf-core rnaseq pipeline described above. Biological replicate transcriptome concordance was assessed using principal component analysis (PCA).

#### 4.4.3. Differential Gene Expression and Gene Set Enrichment Analysis (GSEA)

Differential gene expression analysis and GSEA were performed using the nf-core differential abundance pipeline v1.5.0 using the following parameters: --filtering_min_abundance 4 --filtering_min_proportion 0.50 --gsea_run true --gsea_permute “gene_set” --gsea_nperm 1000. The mouse gene sets v2023.2.Mm from MSigDB were used for GSEA. Differentially expressed genes (DEGs) were defined by a minimum fold-change of 1.25 and FDR < 0.05.

#### 4.4.4. Pathway Analysis with Metascape and IPA

Pathway and Upstream Regulator Analyses on differentially expressed genes were performed using Metascape [57] and QIAGEN Ingenuity Pathway Analysis (IPA) software 24.0.1 (QIAGEN Inc., Germantown, MD, USA) [58].

### 4.5. Statistical Analysis

Statistical analysis was conducted using R software 4.4.1 (R Foundation for Statistical Computing, Vienna, Austria) and GraphPad Prism 10 (GraphPad Software, LLC, Boston, MA, USA). Unless stated otherwise, one-way ANOVA with Fisher’s LSD post hoc test was used to assess differences between multiple groups. Results were considered statistically significant at *p* < 0.05. Data are presented as mean ± SEM.

## Data Availability

The raw data supporting the conclusions of this article will be made available by the authors on reasonable request.
